# Wearable AI to enhance patient safety and clinical decision-making

**DOI:** 10.1038/s41746-025-01554-w

**Published:** 2025-03-22

**Authors:** Arjun Mahajan, Kimia Heydari, Dylan Powell

**Affiliations:** 1 Harvard Medical School, Boston, MA USA; 2 Faculty of Health Sciences & Sport, University of Stirling, Stirling, UK

**Keywords:** Health care, Medical research

## Abstract

Wearable artificial intelligence (AI) technologies show promise in healthcare, with early applications demonstrating diverse benefits for patient safety. These systems go beyond traditional data collection, using advanced algorithms to provide real-time clinical guidance. From infectious disease monitoring to AI-powered surgical assistance, these technologies enable proactive, personalized care while addressing critical safety gaps. However, successful implementation requires careful consideration of technical, operational, and ethical challenges.

In the post-pandemic era, healthcare systems face mounting challenges with managing increasing patient volumes and complexity, leading to persistent issues with preventable adverse events^1^. At the same time, this period has seen remarkable advances in and adoption of digital health capabilities^2^. Wearable artificial intelligence (AI) technologies represent a significant opportunity in health, emerging at this critical juncture and promising to improve consumer or citizen health and safety.

AI-enabled wearable devices, hereinafter referred to as wearable AI, go beyond traditional wearable devices by not only collecting real-time health data, but also using advanced algorithms to analyze multiple types of patient data and provide guidance for clinical care decisions^2,3^.

This article, focusing on provider-oriented applications, examines how such intelligent wearable systems may improve patient safety and clinical care across healthcare settings—from routine monitoring to complex surgical procedures—marking an important shift from devices that merely collect data to those that predict and prevent errors in real time.

## Practical applications of wearable AI in healthcare

The implementation of wearable AI in healthcare spans diverse applications, with several technologies already demonstrating significant impact. Advanced continuous glucose monitoring systems now incorporate AI algorithms that not only track blood sugar levels but predict dangerous fluctuations hours in advance, enabling preemptive interventions for chronic conditions^4^. Similarly, AI-enhanced cardiac monitors utilize machine learning to detect subtle arrhythmias and predict potential cardiac events with high accuracy, significantly improving early detection rates^5^.

A more recent development is the implementation of computer vision and AI-powered camera systems in operating rooms and acute-care settings, including those that can be ‘worn’ by providers during procedures^6^. These systems use deep learning to detect medication errors in real-time, analyzing medication labels, dosages, and administration procedures against patient data and standard protocols. This technology addresses a critical gap in surgical safety, where traditional manual checks may fail amidst intense and multi-directional demands on providers^6^. These technologies, particularly when applied to high-stakes or acute-care settings where individual decisions often swiftly shape outcomes, demonstrate the potential for AI to serve as an intelligent safety net, augmenting human expertise rather than replacing it.

Moreover, in resource-limited settings, novel AI-enabled photoplethysmography (PPG) wearables are now being developed to address critical healthcare gaps, such as in predicting deterioration of dengue fever hours in advance, marking wearable AI’s pivotal and emerging capability to support clinical decision-making in settings where monitoring resources are scarce or with constrained healthcare infrastructure^7^.

Many of these technologies have progressed beyond theoretical concepts, undergoing validation in clinical trials. Some, like certain cardiac and vital sign monitoring systems, have already received FDA approval for clinical use, while others remain in various stages of regulatory review or are being utilized under research protocols^3^.

## Potential opportunities for patient safety and care quality

Wearable AI technologies may help improve patient safety protocols and care quality metrics through multiple mechanisms^8,9^.

AI systems’ predictive analytics capabilities now enable the identification of subtle patterns and early warning signs that precede serious health events. These capabilities are powered by advancements such as *transfer learning* (a technique where insights gained from one dataset or task are applied to improve performance on a different but related task) and *federated learning* (an approach that securely trains AI models using data from multiple sources without actually sharing patients’ raw data, addressing key privacy concerns while enabling collaborative model development).

The transition from intermittent to continuous monitoring represents a crucial advancement in patient surveillance - where uninterrupted data streams reveal previously undetectable physiological trends and variability patterns that static measurements miss, offering deeper insights into disease progression and therapeutic response^10,11^. This capability spans from detecting early sepsis patterns in hospitalized patients to predicting exacerbations in chronic conditions like COPD at home, representing a fundamental shift from reactive to proactive healthcare delivery^10,11^.

Second, AI-powered contextual awareness may enhance the delivery of personalized care by contiuously adapting interventions and therapeutic guidance based on multidimensional and thus more holistic real-time analysis of patient data^12^. These systems can automatically adjust medication recommendations or lifestyle guidance by integrating multiple data streams—from sleep quality, to physical activity patterns, to medication plan adherence—enabling personalized interventions that evolve with each patient’s unique conditions, lifestyle constraints, and preferences, more likely to provide patient-centered care and ensure long-term adoption and usage^2,12^.

Third, the integration of multiple AI wearables is creating intelligent healthcare environments that work in concert to enhance safety. In acute care settings, AI-powered monitoring systems can detect medication errors in surgery and detect and classify bleeding lesions in real-time during colonoscopy, while broadly in everyday care, networks of connected devices can cross-validate readings and decisions to prevent errors and optimize treatment responses^6,13^. These systems have progressed beyond design stages and are now being tested in controlled clinical environments, with ongoing work to establish standards for adoption across diverse healthcare facilities^6,13^.

## Challenges, considerations, and future directions

As the potential of wearable AI in healthcare expands, several critical challenges must be addressed across technical, operational, and ethical dimensions to ensure successful deployment and adoption

### Technical considerations

For wearable AI systems to achieve reliable performance, devices will need to address several critical technical challenges in data collection and processing. Sensors must be able to maintain signal quality and filter out noise from constant movement, poor contact points, and varying environmental conditions^14^. Devices must also ensure consistent and accurate readings regardless of how they are worn or positioned on the body, and across diverse user activities from sleep to exercise^15^. Additionally, the intensive computational requirements of continuous AI monitoring must be balanced against the fundamental constraints of battery life and processing power in compact wearable forms^15^.

### Implementation considerations

The successful implementation of wearable AI systems requires careful consideration of both economic and human factors across the healthcare ecosystem. Beyond the initial hardware costs, healthcare systems must invest in the digital infrastructure needed to integrate these devices with existing medical records systems, while ensuring staff receive adequate technical training to interpret and act on the AI-generated insights^16^. Provider adoption will depend not just on proving clinical value, but on developing streamlined workflows that allow physicians to efficiently incorporate continuous monitoring data into their practice without increasing their already heavy workload^16,17^. Meanwhile, patient engagement requires devices that are not only comfortable and easy to use, but also provide meaningful, actionable feedback that motivates sustained long-term use rather than contributing to alert fatigue or anxiety about health metrics^18^.

### Patient safety and care quality considerations

The integration of wearable AI technologies into clinical settings requires rigorous safety protocols and quality monitoring frameworks to mitigate potential risks to patient care. AI algorithms supporting diagnostic or therapeutic decisions require not only thorough validation processes (i.e., clinical trials demonstrating efficacy and safety), but also clear contingency protocols for system failures, downtimes, or algorithmic errors that could compromise patient safety in critical care scenarios. Continuous post-implementation monitoring is equally essential, with systematic tracking of near-misses, adverse events, and regular quality audits to identify emerging safety concerns. Furthermore, healthcare systems should establish clear accountability frameworks that delineate responsibility among technology providers, healthcare institutions, and clinicians when AI-augmented decisions contribute to adverse outcomes, ensuring appropriate oversight.

### Privacy and ethical considerations

The widespread deployment of wearable AI systems raises critical privacy and ethical considerations that must be carefully balanced against their clinical benefits. While continuous health monitoring generates valuable data for improving care, it requires robust security protocols to protect sensitive personal information during collection and transmission, along with secure integration pathways that meet stringent healthcare data compliance requirements^19^. Healthcare systems must also navigate the complex challenge of aggregating data to improve AI algorithms while preserving individual privacy and patient autonomy—including giving patients meaningful control over how their data is shared and used^20^. Moreover, as with other AI-powered systems, ensuring these systems are trained on diverse and representative datasets is crucial to prevent algorithmic bias and ensure equitable health monitoring across different populations and demographics^21^.

## Conclusion

**Fig. 1:**
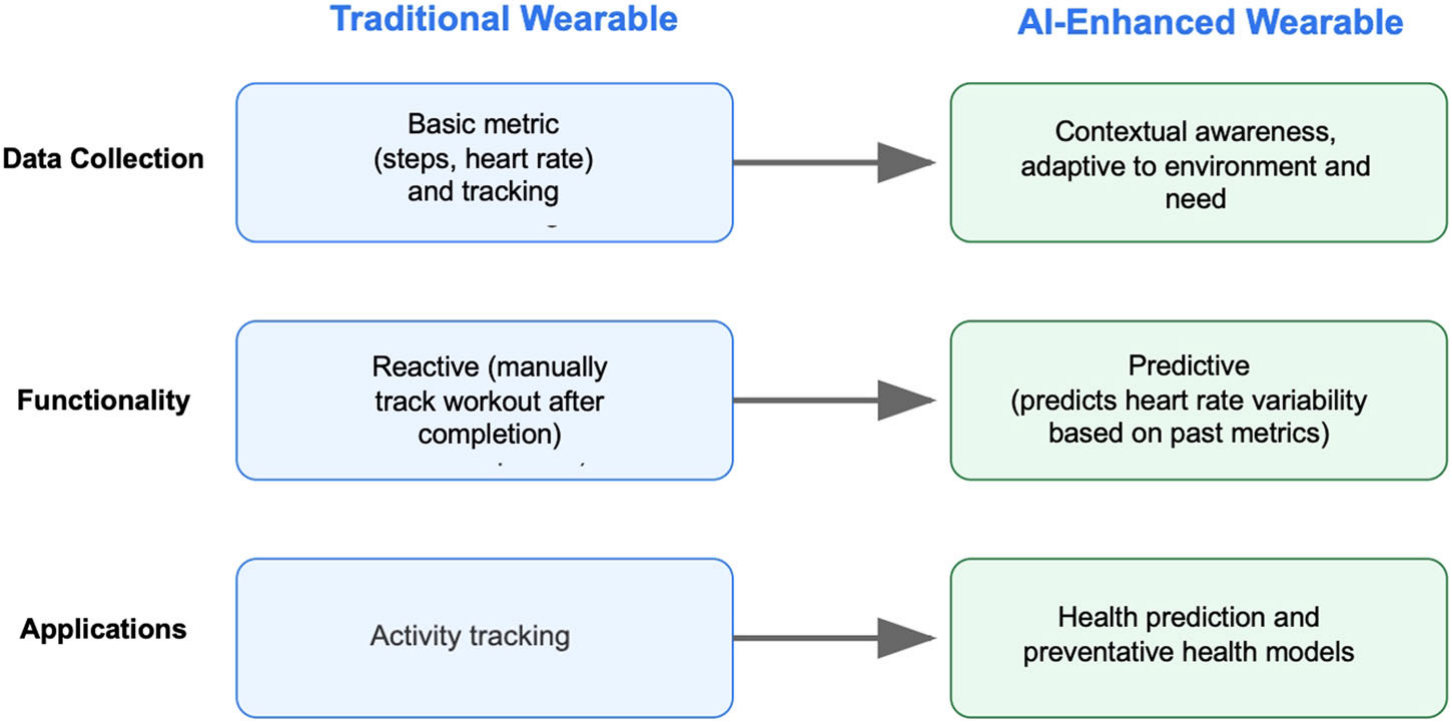
Example functions and applications of AI-enhanced wearable devices.

While wearable AI technologies represent a transformative force in healthcare, realizing their full potential requires addressing critical technical, operational, and ethical challenges through collaborative innovation. With thoughtful implementation and continued technological advancement, these systems promise to fundamentally reshape healthcare delivery by enabling truly proactive, personalized, and patient-centered care^22^ (Fig. 1).

## Data Availability

No datasets were generated or analysed during the current study.
